# Pain Management in Cancer Patients With Artificial Intelligence: Narrative Review

**DOI:** 10.1155/sci5/6888213

**Published:** 2025-04-22

**Authors:** Golnar Ghane, Raoofeh Karimi, Amir Mohammad Chekeni, Mahdi Darvishi, Reza Imani, Fatemeh Zahra Vafaeinezhad

**Affiliations:** ^1^Medical Surgical Department, School of Nursing and Midwifery, Tehran University of Medical Sciences, Tehran, Iran; ^2^Medical Surgical Department, Nursing and Midwifery School, Student Research Committee, Tehran University of Medical Sciences, Tehran, Iran; ^3^Pharmacy Department, Jamia Hamdard University, New Delhi 110062, India; ^4^School of Medicine, Tehran University of Medical Sciences, Tehran, Iran

**Keywords:** artificial intelligence, cancer, pain, pain management

## Abstract

**Background:** Pain is a significant symptom in cancer patients that is frequently not effectively treated, and managing it is seen as a crucial aspect of caring for these patients. This severe pain frequently causes a significant disturbance in their quality of life. At present, there are different challenges in utilizing a range of pharmacological and nonpharmacological treatments for managing pain in cancer patients. Recent technological advancements, particularly in artificial intelligence, have improved the management of pain in cancer patients. Artificial intelligence and its algorithms offer potential solutions for pain relief in cancer patients with reduced side effects.

**Study Design:** The current review aimed to assess the validity of studies on using artificial intelligence in pain management for cancer patients. Four databases have been used to review all published studies from the start of 2023: PubMed, Scopus, Web of Science, and Google Scholar. The search mechanism for articles was mainly using valid and mesh-based keywords, asking experts, and reviewing the literature and including “Pain,” “Pain management,” “Cancer,” and “Artificial intelligence.” During the initial search, a total of 450 articles were found, and after considering the inclusion and exclusion criteria and reviewing the abstract and content of the articles, 15 articles were finally included in the study.

**Results:** AI-based solutions can provide individual pain relief plans. When AI analyzes large patient data such as physiological signals, responses to treatment, and symptoms of patients who have been diagnosed with pain, it is possible to accurately adjust therapeutic measures.

**Conclusions:** AI enables healthcare providers to offer timely care and assistance to cancer patients through remote monitoring and telehealth services, even when they are not physically present. Despite the presence of hurdles such as ensuring ethical AI practices and protecting data privacy, the integration of AI in oncology pain management brings optimism for the future.

## 1. Introduction

The International Association for the Study of Pain (IASP) defines pain as a sensory experience linked to tissue damage, either current or possible [1].

Pain caused by cancer is a highly distressing symptom for those with cancer, particularly as the disease progresses [2]. Pain may result from cancer spreading to bone, invading soft tissue, or putting pressure on nerves, or from cancer treatment such as mucositis, musculoskeletal pain, postoperative pain, dermatitis, or enteritis [3]. In terms of its causes, cancer pain is caused by multiple factors and is defined by different pathophysiological mechanisms involving nociceptive, neuropathic, and combined mechanisms [4].

According to the International Society for the Study of Pain, cancer-related pain is a significant issue for the over 10 million individuals who are diagnosed with some form of cancer annually. Although cancer pain is not always a certainty for every patient, it is a frequent occurrence [5] with 64% of those with metastatic cancer and 59% of those receiving anticancer therapy reporting it [6]. Furthermore, more than one-third of cancer patients view pain as an intolerable aspect of their condition [5].

Pain's presence and intensity have significant clinical implications since they impact health-related quality of life (HRQOL). Inadequate pain management results in diminished communication between healthcare providers and patients and reduces patient satisfaction. It also results in emotional anguish and decreased participation in activities and social connections. Increased pain reporting by the patient leads to better quality of life and adherence to treatment [6].

Pain in cancer patients is frequently not adequately treated. The frequency of pain differs based on the seriousness of the illness and cancer, yet research did not find a notable discrepancy in pain levels between solid tumors and hematological malignancies [6]. Patients with cancer noted that pain disrupts their ability to focus and carry out daily tasks [5].

Pharmacological and nonpharmacological methods are used to manage pain in cancer patients. Treatment choices consist of common options for managing physical pain: opioids like oxycodone, morphine, hydromorphone, and fentanyl, as well as anticonvulsants such as gabapentin and pregabalin for neuropathic pain [4]. Although cancer patients receive radiological, surgical, and pharmacological therapies, studies indicate that a significant number still suffer from excessive levels of pain [7].

In pain management, it is only feasible to continuously monitor patients throughout the day with the use of technology-based solutions, and it is important to verify the accuracy of the observations and findings. The observer might not evaluate the patient's pain accurately because they lack training or have personal biases. Don't forget to also focus on social and interpersonal connections. The development of automatic pain detection systems is a result of these reasons, as they are able to identify the presence and intensity of pain, as well as analyze physiological and behavioral responses. The area of artificial intelligence (AI) is concentrated on developing models and algorithms that can detect and recognize emotions and feelings, including pain [1].

Visual analog scales (VAS), the McGill Pain Questionnaire, and numerical rating scales (NRS) are frequently regarded as the top choice for assessing patient-reported outcome measures (PROs, self-reports, or PROMs). These strategies cannot be used for infants, individuals with delirium, those under sedation and mechanical ventilation, or individuals with dementia or developmental and intellectual disabilities [8].

AI imitates the operation of the human brain by utilizing various symbolic and statistical techniques for learning and reasoning. It consists of machine learning (ML), computer vision (CV), fuzzy logic (FL), and natural language processing (NLP). The goal of utilizing AI is to create dependable pain evaluation techniques. In general, these techniques are referred to as automated pain assessment (APA) [9].

In recent years, a growing number of research studies have utilized interventions based on AI to enhance pain diagnosis, prediction, and self-management. However, no one has thoroughly examined how AI can be used to improve the evaluation and treatment of pain. Because of the significance of this matter, the current research was carried out to examine the story of pain control in cancer patients with the use of AI.

## 2. Methods

This review study was conducted to assess the validity of studies conducted in the field of pain management in cancer patients with AI. All published studies from the beginning to 2023 were reviewed in four databases: PubMed, Scopus, Web of Science, and Google Scholar. The search mechanism for articles was mainly using valid and mesh-based keywords, asking experts, and reviewing the literature, including “Pain,” “Pain management,” “Cancer,” and “Artificial intelligence.”

**Figure 1:**
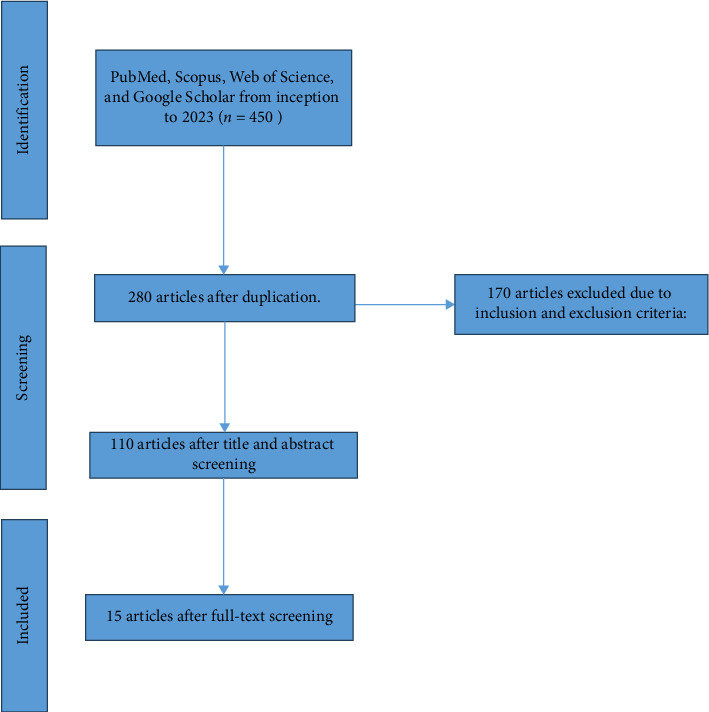
Flowchart of articles' selection process.

After customizing the search formula for each database, the researchers searched in each of these databases. In order to increase the sensitivity of the search and to ensure that all related articles are entered, the researchers checked the references of the final articles entered as well as the previous systematic review articles. During the initial search, a total of 450 articles were found. The criteria for inclusion in the study include articles published in reputable scientific research journals, having English or Persian language, access to the full text of articles in databases, and the connection between the titles of the articles and the title under investigation. Case report studies, case reports and studies that reported the main outcomes of the study in an ambiguous manner, studies whose text was not available after correspondence with the responsible author, studies that were retracted after review, and gray studies such as Conference articles and theses were excluded from this study. 15 articles were selected for final review by analyzing their abstracts and content and using predetermined inclusion and exclusion criteria (Figure 1).

## 3. AI in Pain Assessment

### 3.1. Camera-Based Approaches

Pain control is one of the important and key aspects among health workers in clinical settings, which enables better treatment and management. Considering the limitations that traditional methods have in pain assessment (Table 1), camera-based methods emerged as noninvasive and beneficial methods, which have been supported in several studies of their effectiveness. For example, in the study by Tavakolian and Hadid, they presented a new 3D depth model for dynamic spatiotemporal representation of faces in videos and automatic estimation of pain intensity from faces [10]. The results of the study by Wu et al. also confirm the practical application of deep learning-based APA in critically ill patients [11]. In the study by Reichard et al., a method for classifying pain based on mimicry descriptors was presented and the Facial Action Coding System (FACS) was used [12]. The results of the review conducted by Salama et al. also indicate the key role of AI/ML in predicting pain in cancer patients undergoing treatment [13].

#### 3.1.1. Facial Gestures (Grimace, Mouth Opened, and Raising of Eyebrows)

Using a program with AI for face recognition, the smartphone camera captures facial movements. Facial expressions are a key in expressing emotions and recognizing pain. Hence, the majority of research has concentrated on this approach. The introduction of the NBC-McMaster database, the first public database for pain recognition, further strengthened this trend by offering detailed annotations along with facial images. Alternatively, other stress-inducing factors can induce a condition like pain, resulting in simulated facial expressions of pain [16].

Facial expressions are a helpful tool for determining pain levels as pain tends to be connected with natural facial movements. Simple pictures or videos of faces were frequently utilized in this context. The FACS is a technique used to describe and study observable facial expressions. It consists of 44 basic units known as action units (AUs) that correspond to the muscle activation and evaluate movements. FACS offers a manual with a collection of pictures and drawings to assist in scoring these AUs, offering guidance. Facial animation software is utilized in psychology, sociology, and communication studies, as well as for facial palsy assessment.

Convolutional neural networks (CNN) are used for image classification and object recognition tasks. CNNs are a form of feed-forward artificial neural network (ANN), in which there are no loops created by connections between nodes. They perform the identical role to the retina by transforming input information into output. It comprises multiple layers, starting with the convolutional layer, followed by a nonlinear layer and several pooling layers before reaching the fully connected layer (flattening). Classification is carried out in the final layer by using the features obtained from earlier layers and different filters. The CNN structure may differ, but the VGG 16 architecture serves as a model for constructing CNNs. It is made up of 16 convolutional layers with a size of 3 × 3 and several filters. VGGFace is frequently utilized in pain research. This model has over 3 million face images of over 9000 individuals and is a version of the VGG16 and VGG19 models, utilizing a vast dataset of facial images (VGGFace2). The VGGFace model is suitable for tasks such as face recognition, face verification, and emotion recognition. In this regard, Zamzmi et al. used a novel lightweight convolutional neural network as well as other popular CNN architectures to assess infant pain. They suggest that automated infant pain detection using CNNs is a suitable and more efficient alternative to traditional assessment [17]. Hosseini et al. also used a deep CNN (DCNN) model through facial expressions to predict pain intensity, which yielded promising results with high accuracy [18]. Bargshady et al. also used a Visual Geometric Group Face Convolutional Neural Network (VGGFace CNN) to classify pain intensity from face [19].

Various kinds of ANNs can also be applied to tasks involving image processing. For instance, deep residual networks (ResNets) enable enhanced performance. Additionally, generative adversarial networks (GANs) are a form of ANNs comprising two neural networks: one creating images and the other determining authenticity by comparing them to real images. The autoencoder is another type of neural network architecture utilized for compressing and reconstructing images. U-Nets are another type of CNN model that is employed for tasks involving the segmentation of images. For example, Monwar and Rezaei used efficient video analysis techniques and ANNs to classify painful and painless faces and pain recognition [20]. Shuang et al. also used ResNet50 to build an automatic pain expression recognition model in elderly patients with hip fractures, which had high accuracy [21]. Wang and colleagues also used a generative deep learning method to predict pain in patients in the ICU, which had an accuracy of 97.2% [22].

RNNs are neural networks created for the purpose of handling sequential data like time series information or natural language text, enabling the transfer of information between different stages of the sequence. LSTM, a form of RNN, excels at recognizing and retaining long-term relationships in sequential data. LSTMs have been applied to different tasks like processing natural language, recognizing speech, and predicting time series data. BiLSTM is a type of network that includes two LSTMs, with one handling input in the forward direction and the other handling input in the backward direction. They have the ability to examine data including facial expressions, body language, speech, and heart rate (HR). CNNs excel in extracting features from images, whereas LSTM networks excel in handling sequential data. Thus, a hybrid neural network is suggested for pain research, which integrates both a CNN and an LSTM network. Cascella et al. recently employed CNN and LSTM to categorize facial emotions from diverse openly accessible datasets [9, 16]. The results of the study by Rojas et al. showed that the BiLSTM model had the highest accuracy in assessing pain in patients who were unable to speak [23].

CNN and ANN are both types of neural networks used in ML, but they have different architectures and are suited for different types of tasks. When it comes to facial expression recognition, CNNs are generally considered to be more effective due to their ability to capture spatial hierarchies and patterns in images. The differences between CNN and ANN in the context of facial expression recognition are outlined as follows (Table 2).

#### 3.1.2. Which Technique Is Better for Facial Expression Recognition?

For facial expression recognition, CNNs are generally considered better than ANNs because:

• CNNs can automatically learn relevant features from raw images, which is crucial for capturing the subtle changes in facial expressions.
• The hierarchical structure of CNNs allows them to capture complex patterns and maintain the spatial relationships between different facial features.
• CNNs are more efficient in terms of the number of parameters and computational resources, especially for high-dimensional image data.

In summary, CNNs are the preferred choice for facial expression recognition due to their ability to handle image data effectively and their superior performance in capturing the intricate details necessary for accurate expression classification.

#### 3.1.3. Example of “Pain” vs. “No-Pain” States in Camera-Based Approaches

In the context of pain management in cancer patients, camera-based approaches often involve the use of facial recognition and analysis to distinguish between “pain” and “no-pain” states. These systems typically rely on the detection of facial expressions associated with pain, which can be challenging due to the subjective nature of pain and the potential for variations in individual pain responses [24].

##### 3.1.3.1. Facial Expression Analysis

• Pain State: The system detects specific facial expressions that are commonly associated with pain, such as brow lowering, eye squeeze, and mouth stretch. These expressions are often quantified using AUs from the FACS.
• No-Pain State: In the absence of pain, the system would not detect these pain-related facial expressions. The patient's facial expressions would be more neutral or reflective of other emotions or states that are not indicative of pain [16].

#### 3.1.4. Guidelines for Differentiating Pain States

Several studies and research groups have developed guidelines and frameworks for using camera-based approaches to differentiate between pain and no-pain states. These guidelines often involve as follows:

1. Standardized Pain Scales:
• NRS: Patients rate their pain on a scale from 0 (no pain) to 10 (worst possible pain).
• VAS: Patients indicate their pain level by marking a point on a line where one end represents no pain and the other end represents the worst possible pain.
2. FACS:
  This system provides a standardized method for measuring and describing facial expressions. It breaks down facial movements into individual AUs, which can be correlated with specific emotions or sensations, including pain.
3. Automated Facial Analysis Software:
  Software like the Computer Expression Recognition Toolbox (CERT) or the Automated Pain Monitor (APM) can automatically detect and analyze facial expressions in real-time. These tools are trained to recognize patterns associated with pain and can provide objective measures of pain intensity [1].

#### 3.1.5. Incorporation Into Pain Management

These camera-based approaches can be integrated into pain management protocols for cancer patients in various settings:

1. Home Care:
  Patients can use smartphone apps equipped with facial recognition software to monitor their pain levels at home. This can help in adjusting pain management strategies based on real-time data.
2. Inpatient Settings:
  In clinical settings, camera-based systems can provide continuous monitoring of patients' pain levels, allowing healthcare providers to intervene promptly and adjust treatment plans accordingly [25].

Camera-based approaches offer a promising avenue for objective pain assessment in cancer patients. By distinguishing between “pain” and “no-pain” states through facial expression analysis, these systems can enhance the accuracy and timeliness of pain management interventions. However, it is important to note that these approaches should be used in conjunction with other pain assessment methods and patient self-reports to ensure comprehensive and effective pain management [26].

### 3.2. Contact-Sensor Approaches

The most commonly used sensors for evaluating pain were found to be EDA, ECG, and EEG.

#### 3.2.1. Physiological Measures

Electrodermal activity (EDA), surface electromyography (sEMG), respiration (RESP), oxygen saturation (SpO2), blood pressure (BP), electrocardiogram (ECG), movement (MOVE), skin temperature (SKT), pupillary response (PUPIL), and photoplethysmography (PPG) are all physiological measurements that can be used in research and monitoring.

EDA is a widely utilized physiological signal in psychiatry and psychology research. EDA, also known as galvanic skin response or skin conductance response, measures changes in the electrical resistance or conductivity of the skin. This signal reflects changes in sweat gland activity, which is modulated by the sympathetic nervous system. Increased sweating in response to stimuli leads to a decrease in skin resistance and an increase in skin conductance. EDA is commonly recorded from the palms and soles of the feet due to the higher density of glands in these areas [27].

Another commonly used method to evaluate pain is electrocardiography (ECG). The ECG signal shows the heart's contracting activity. The autonomic nervous system is responsible for controlling the internal functions of the body, such as the cardiovascular system, without conscious effort. The most crucial pain-related parameters of ECG signals are HR and heart rate variability (HRV). Pain is linked to higher HR and lower HRV [27].

Electromyography (EMG) serves as an alternative method for pain assessment. This method evaluates muscle electrical activity on the skin's surface. No specific muscle serves as a pain indicator, but heightened trapezius activity is associated with stress in affective computing, whereas increased zygomaticus and corrugator muscle activity is linked to reacting to negative images. The trapezius muscle, zygomaticus major, and corrugator supercilii muscles were utilized for EMG measurement in the examined articles [27].

PPG is another reliable technique for detecting alterations in blood volume and is frequently utilized in transmission (like a clip on an earlobe or finger) or reflective mode (as seen in a wristwatch). The finger or earlobe is where most often seen. The PPG signal allows for the determination of metrics like HR, SpO_2_, and respiratory rate.

SKT is an additional metric for detecting pain. The skin's resistance decreases when sweat is produced. Because there is a greater concentration of glands in the palms and soles, this characteristic can be assessed in those regions.

Other signs like RESP rate, SpO_2_ levels, BP readings, MOVE patterns, and changes in pupil size (PPIL) are also utilized to assess pain. Respiratory alterations frequently happen due to pain and can be monitored with the use of a stretchy band placed around the chest. Changes in breathing, oxygen levels, and BP are linked to pain. Painful stimulation also led to a decrease in the size of pupils, also known as dilation.

Most contact-based sensors have drawbacks: movement can cause errors and the signals can be influenced by changes in the sensor's contact with the skin, such as adhesive electrodes becoming loose and needing reattachment [27, 28].

#### 3.2.2. Neurological Measures

The human brain plays a crucial role in responding to stimuli, as neural signals are interconnected with intricate functions like sensory and motor coordination. In research, two different neurosensors were employed for pain evaluation: electroencephalography (EEG) and functional near-infrared spectroscopy (fNIRS).

EEG was the most frequently used method for assessing pain. EEG is used to monitor the electrical signals in the brain, which are analyzed to evaluate levels of pain. EEG is the most commonly used method to evaluate cognitive functions like attention and alertness. EEG data are segmented into five categories: delta, theta, alpha, beta, and gamma. Evaluations make use of the variations in the intensity of each of these bands.

Another method for evaluating pain using neurosensors involves assessing the activity of various brain areas through fNIRS, which detects levels of oxygenation (HbO) and deoxygenated hemoglobin (HbR) in the brain cortex [27].

### 3.3. Audio Approaches

Vocal reactions to pain, like nonverbal cries, groans, or shouts, made by individuals in pain can act as signs of pain in humans.

The earliest projects in the audio sector can be traced back to 1990, with a primary focus on identifying pain in infants based on their cries. One of the main obstacles in analyzing audio data is distinguishing the desired sounds from any interference coming from sources such as medical equipment, individuals, or surrounding events. This approach also involves studying breathing patterns and vocal expressions to evaluate levels of discomfort.

**Figure 2:**
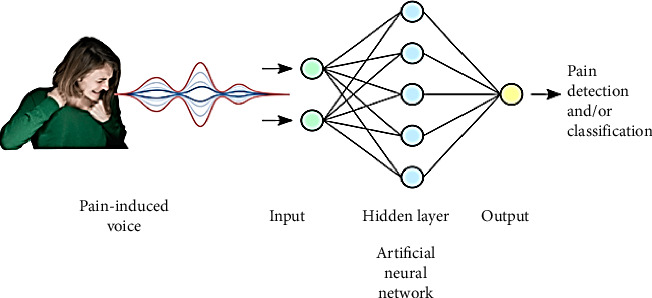
Audio approaches in pain management.

At present, there is a strong emphasis on incorporating AI algorithms in audio events and voice recognition. Keyword spotting (KWS), wake-up words (WUW), and speech command recognition (SCR) are crucial methods in speech processing that allow machines to identify spoken words and react appropriately. This approach is widely used in assessing pain in infants (Figure 2) [8].

### 3.4. Multimodal Approaches

Using diverse assessment techniques can enhance each other, resulting in increased specificity and sensitivity, ultimately leading to better outcomes. However, it results in enhanced flexibility and accessibility. In medical environments, a procedure might not be possible for reasons such as facial injury. A multimodal system might be able to make up for the lack of one or multiple methods and still offer a valuable evaluation [27, 28].

## 4. Personalized Treatment Plans

It involves utilizing an individual's specific traits to provide therapy. The concept of personalized treatment suggests that doctors can use individual characteristics to make treatment decisions more effective. Identifying key traits that differentiate individuals plays a crucial role in advancing personalized treatment strategies. This solution identifies the optimal treatment for each individual based on specific circumstances [29]. An individualized treatment approach in patient care, where personal factors such as genetic profile are taken into consideration in clinical decision-making, aims to provide the most suitable treatment to each patient at the appropriate time [30].

Personalized treatment is now being implemented in cancer, cardiovascular diseases, infectious diseases, and transplant medicine. Aligning individuals with the most suitable treatment based on their unique characteristics has the potential to enhance the treatment's efficacy for them, leading to improved overall effectiveness [29]. A pain management treatment plan based on the genetic characteristics of individual patients is recommended as personalized treatment. Tailored treatment may enable doctors to more effectively manage patients' pain and establish a new approach to treating acute postoperative pain [31].

## 5. Predictive Analytics and Telehealth Integration

### 5.1. Predictive Analytics

Advancements in computational power and ML have resulted in notable breakthroughs in the medical field. AI algorithms can now identify pneumonia from chest X-rays and diabetic retinopathy from fundoscopic images with performance that can match or surpass clinician diagnostic skills. Predictive analytics has been in a prime position to analyze the vast amounts of data generated by electronic health records (EHRs). Predictive analytic algorithms that have been made public have demonstrated the ability to forecast and occasionally stop significant occurrences, like readmissions related to heart failure, chronic obstructive pulmonary disease, and neonatal sepsis [32].

#### 5.1.1. Predictive Analytics in Cancer

Large amounts of data and predictive analytics can greatly enhance risk assessment, particularly in data-heavy areas such as oncology. A field abundant in data, such as oncology, appears to be a suitable fit for predictive analytics [32]. In the past few years, there has been a significant increase in the field of AI within oncology. AI solutions have been created to address various challenges related to cancer. Healthcare facilities, hospitals, and tech firms are creating AI tools to help with medical decision-making, enhance cancer treatment accessibility, and boost clinical productivity in order to provide secure, top-quality cancer care. AI in the field of oncology has shown high levels of accuracy in tasks such as analyzing images, making predictions, and providing precise treatment options for cancer patients [33]. Predictive analysis tools use historical patient data to automatically project future health results for individuals or groups. As the volume of data in oncology, including EHR, radiology, genomic, and other types, has grown, there have been multiple potentially applicable use cases [32].

#### 5.1.2. Predictive Analytics in Oncology: Challenges

Despite the necessity for improved predictions of life expectancy, acute care utilization, adverse effects, and genomic and molecular risk, there are few instances of predictive analytics being utilized in the field of oncology. We believe that existing predictive analytic interventions have the potential to fill significant gaps in risk stratification strategies in the field of oncology. Nevertheless, healthcare professionals, technology experts, and decision-makers need to tackle the obstacles related to research, technology, and regulations that hinder the use of analytics in the field of oncology [32].

Risk assessment in the field of cancer treatment is hampered by the absence of necessary prognostic details, the requirement for laborious manual data entry, limited access to thorough data, and in certain instances, an excessive dependence on clinical judgment. Lack of accurate identification of high-risk patients with cancer can result in either overly aggressive end-of-life treatment or unnecessary use of acute care. Additionally, the evaluation of prognostic factors such as performance status can be influenced by variations and prejudices among clinicians [32].

#### 5.1.3. Predictive Analytics and Molecular Markers

Recent research is exploring how tumor genomic and molecular biomarkers can predict the success of treatments and patient outcomes. Currently, there are minimal studies that have tried to combine genomic and clinical biomarkers in predictive models. With the introduction of molecular-based treatments and immunotherapy, cancers must be categorized more specifically according to these molecular indicators. Newer systems like the updated Katagiri have integrated molecular features of cancer in the prognostic model, including the hormone status of breast cancer and molecular targets of non–small-cell lung carcinoma. Considering genetic or molecular expression in predictive analytics may represent the next frontier in predicting clinical outcomes [34]. Combining information from clinical, genetic, and molecular predictive factors could lead to a more accurate and detailed risk assessment in oncology, paving the way for precise precision medicine [32].

#### 5.1.4. Radiomics

The growing area of radiomics is an example of how predictive analytics models are starting to be utilized in the field of oncology. Radiomics is the study of tumor characteristics through quantitative data analysis of scans. These traits can help in identifying, describing, and keeping track of solid tumors. Computer-aided detection can be used to detect cancerous lung nodules on CT scans and prostate lesions on MRI scans, and it might also be useful for automating tumor staging [32].

#### 5.1.5. Pathology

Pathology is a crucial area in the practice of oncology that is ready to see advantages from predictive analytics. There are many models developed to predict outcomes and anticipate therapy responses based on tumor pathologic characteristics for certain diseases. The 21-gene recurrence score and the 70-gene recurrence score are commonly used in clinical practice for determining the need for chemotherapy in patients with early-stage breast cancer [32].

#### 5.1.6. Predictive Computer-Based Models

**Figure 3:**
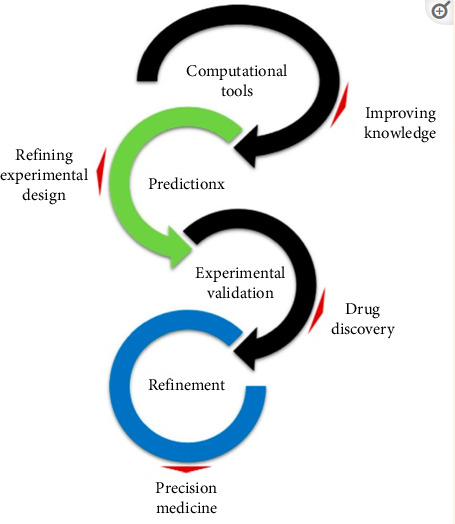
Refinement process of computer-based models with new incomes.

Experimental and computational models of cancer have offered a valuable option to studying patients directly, helping to address concerns related to experimental design, ethics, finances, and animal welfare. The results can be related to in vivo studies to enhance the models and aid in designing predictive-focused experiments (Figure 3) [35].

#### 5.1.7. Utilize Predictive Analytics in Pain Management

**Figure 4:**
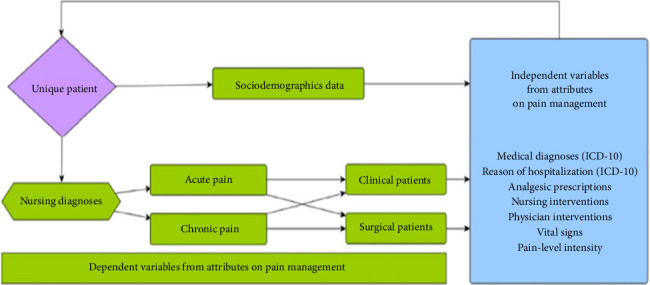
Predictive models in pain management.

Dealing with pain is seen as a worldwide issue that needs a combined effort due to its complex nature, involving various aspects such as biology, culture, development, psychology, empathy, and situational factors. By examining extensive data on pain treatment from vast databases like EHRs and information systems, it is feasible to offer real-time guidance on care management and identify patient characteristics through their clinical data repositories, leading to more precise decisions in this area in Figure 4 [36].

### 5.2. Telehealth

Telehealth is widely characterized as a way for patients and providers to have virtual, two-way communication. Telehealth can involve techniques like audio-only, video-consultation, and telemonitoring, which may take place in a synchronous, asynchronous, or blended manner [37]. Telehealth care involves using telecommunication technologies to offer health education, consultation, assessment, and ongoing monitoring to cancer survivors remotely [38]. A novel method using IT connects healthcare facilities and patients in distant locations through telecommunication [39].

#### 5.2.1. Telehealth Applied to Cancer Patients

Improvements in cancer diagnosis and treatment have led to a rise in cancer survival rates. However, cancer survivors often face issues related to their disease or treatment, resulting in a lower quality of life. Different telehealth interventions have been widely used in medical care, proving to be cost-effective, highly satisfying for patients, and well-accepted by health professionals. Telehealth interventions are a successful alternative for enhancing the quality of life in cancer survivors [38].

Successful provision of cancer care through telehealth necessitates a well-thought-out system that considers various resources such as patients, healthcare providers, and clinic/cancer center resources before, during, and after the treatment. Utilizing telehealth for cancer care involves more than just substituting in-person appointments with audio or video consultations; it necessitates thorough program design and proactive education for patients, providers, and healthcare facilities. Even though telehealth seems to enhance fairness in accessing healthcare, persistent challenges related to poverty, underinsurance, limited technology and broadband access, and health literacy continue to hinder equitable care access in low-income areas [37].

#### 5.2.2. Telehealth in Cancer Pain Management

In patients with cancer pain, using telemedicine strategies can improve clinical care and make better use of resources. This method has the potential to play a crucial role in enhancing patient satisfaction and enhancing health results [40].

Nevertheless, addressing pain associated with cancer usually necessitates a multifaceted and varied strategy. Telemedicine has the potential to provide a range of options for reassessing paths of treatment, such as managing cancer pain. However, even with the increasing utilization of telehealth approaches, there is still a lack of scientific proof to create care plans [40]. Currently, there is insufficient evidence regarding the potential uses of telemedicine in helping cancer patients manage pain [9].

### 5.3. Telehealth and Predictive Analytics Integration

The use of predictive modeling is growing in significance within the healthcare industry. A thorough grasp of acquired models and their predictions is essential for the advancement and eventual approval of these systems. The designed viewer concept and its application help to enhance understanding and insight into the predictive modeling development process within the telehealth field [41].

The application of predictive models is a significant change in the field of medicine. The advantages of AI and its subdivisions like ML aim to improve patient care, while also impacting organizational procedures and healthcare systems. When designing care pathways, AI is a useful tool that can enhance hospital processes by pinpointing key tasks and meeting the patient's requirements effectively. It was recently shown that AI techniques like NLP models can effectively prompt early intervention for unmanaged pain and other symptoms in palliative care [40].

Additional experience is required to address uncertainties and determine the optimal approaches for different patient groups in adapting care models based on available resources. It is clear that telehealth methods should be integrated into regular clinical practice for managing cancer pain [9].

## 6. Opioid and Nonopioid Approaches

Management of pain in cancer patients includes both medication and nonmedication treatments. Nonmedication treatments are a vital component of a holistic pain management strategy. Procedures and interventions for treatment range from mind-body practices, acupuncture, massage therapy, and music therapy to pharmacologic options like opioids, nonopioids, and adjuvant drugs (Table 3) [3, 42].

### 6.1. Opioids

Opioids are listed in the WHO model list of essential medicines for acute and cancer pain, palliative care, and treating opioid dependence [43, 44]. Opioids like oxycodone, morphine, hydromorphone, and fentanyl [3] are vital for general anesthesia and perioperative pain management. Their significant pain-relieving effectiveness makes them essential in perioperative care [45]. Opioids have mainly been studied in individuals with moderate-to-severe cancer pain, and they efficiently decrease pain in this group, with known side effects [46]. They are the cornerstone of managing pain in cancer patients and those nearing the end of life [47]. Patients with moderate-to-severe pain caused by cancer or cancer treatment should receive opioids unless there is a reason not to do so [45]. They are known for causing short-term negative effects and have the possibility of causing negative effects in the long term for both patients and society [45]. They ought to be started on an as-needed basis at the lowest effective dose to reach satisfactory pain relief and patient objectives, while also conducting regular evaluations and adjustments [46]. Opioids are essential and suitable for patients with advanced cancer and uncontrollable pain. Nevertheless, they are not suitable for the majority of cancer survivors who do not have active cancer. Due to being exposed to opioids while undergoing cancer treatment, cancer survivors are at an increased risk for opioid misuse. Excessive opioid usage can lead to issues not only in the medical realm but also in mental health, family dynamics, financial burden, and opioid abuse disorder, particularly amidst the ongoing opioid epidemic [3].

Chronic pain and breakthrough cancer pain are prevalent among individuals with various forms and levels of cancer. Commencing treatment with the smallest amount of immediate-release opioids like oxycodone or hydromorphone is crucial. Morphine sulfate is frequently prescribed for breakthrough cancer pain and may be combined with paracetamol or nonsteroidal anti-inflammatory drugs (Table 4) [8, 47].

There are many factors to consider when it comes to the way opioids are administered. Delivery of opioids like a fentanyl patch through the skin. Opioids can also be given directly into the epidural or intrathecal space, which directly affects the dorsal horn of the spinal cord. In certain instances, a surgically implanted intrathecal pump may deliver opioids continuously to manage chronic pain.

Opioid dosage should be determined according to the treatment objectives of each patient, taking into account their pain management goals as well as their ability to tolerate side effects. Beginning opioid treatment in patients who have not previously been exposed to opioids should involve starting at the lowest possible dose and adjusting it based on the patient's reaction and any negative effects. If the patient is able to take oral medications and is not experiencing severe pain, it is best to give them a short-acting opioid in tablet form. The majority of oral medications reach their peak effect at 60 min, while most IV medications reach their peak effect at 15 min [47].

#### 6.1.1. The Role of Predictive Analytics in Opioid Crisis Prevention

Due to the opioid crisis, it is crucial to accurately assess overdose risk and enhance knowledge on providing effective treatments and interventions for individuals with opioid use disorder to minimize overdose risk. Rises in opioid overdoses from pharmaceuticals highlight the importance of supporting prescribers with these tools for prevention planning. A thorough public health response based on evidence is necessary to lessen the negative effects of opioid use. Expanding current interventions, treatments, and resources necessitates a focused strategy to determine the best combination and level of interventions. Although there is proof that some interventions can reduce the risk of overdose, further research is required to determine the most effective way to implement these interventions. Recently, there has been a growing fascination with employing ML techniques to create prediction models. These techniques are frequently touted as being more effective than conventional regression because of their reliance on data and capacity to recognize patterns in data (Table 5) [44].

### 6.2. Nonopioids

Nonopioids like aspirin, acetaminophen (paracetamol), and NSAIDs are utilized for treating mild cancer pain, unlike severe and acute pain. Acetaminophen possesses both fever-reducing and pain-relieving characteristics [42]. It is highly tolerated and lacks the renal, GI, cardiovascular, or bleeding risks seen with NSAIDs. Acetaminophen is often mixed with various opioids to achieve a combined analgesic effect or for the sake of convenience. Reports of liver damage with paracetamol are on the rise, especially when taken in doses exceeding 4 g/day [47].

#### 6.2.1. NSAIDs

This set of medications produces pain-relieving effects on both the central and peripheral nervous systems [42]. Additionally, they can be combined with opioids as a supplementary method for pain management to ensure effective pain relief without interfering with cognitive function or inducing sedation. Nonetheless, the use of NSAIDs comes with various risks, including renal impairment, GI bleeding or perforation, thrombocytopenia, bleeding disorders, and cardiac toxicities [47]. Combining NSAIDs with acetaminophen is beneficial, as the effectiveness of the mixture surpasses that of each individual drug [42]. Cancer patients could face an increased likelihood of experiencing negative effects from NSAIDs due to their cancer treatments or the disease itself, which may already make them more susceptible to such problems [47].

#### 6.2.2. Anticonvulsants

Anticonvulsants like gabapentin and pregabalin might help individuals with neuropathic pain and have the potential to lower the need for opioids, resulting in fewer negative effects [47].

#### 6.2.3. Antidepressants

Medications like duloxetine and venlafaxine, which are serotonin–norepinephrine reuptake inhibitors, as well as tricyclic antidepressants like amitriptyline, desipramine, imipramine, and nortriptyline, are options for managing neuropathic pain [47].

## 7. Adverse Effect

The management of pain in cancer patients, whether through medication or other methods, may have negative effects. Addressing these negative impacts is crucial for cancer care as they can greatly affect the patient's overall well-being. Opioid-Related Adverse Effects: Opioid drugs are often given for intense cancer-related pain. Nevertheless, they may result in negative outcomes like nausea, constipation, and itching [3].

Different AI technologies have been utilized in multiple facets of opioid usage. Further research is required in this area. In research on opioids, AI techniques such as Ensemble algorithms, NLP, deep learning algorithms, instance-based algorithms, regularization algorithms, regression algorithms, Bayesian algorithms, decision tree algorithms, and clustering algorithms were employed [48].

## 8. Challenges in Pain Management

Barriers to effective pain management can be attributed to healthcare professionals lacking adequate knowledge of pain management, inadequate pain assessment, and apprehension about the use of analgesic medications [49, 50]. Due to fears around pain medication, cancer patients may involuntarily impede the successful treatment of their pain, resulting in insufficient pain relief and negative emotional consequences [50]. Despite improvements in cancer treatment, many cancer patients continue to lack proper pain management, leading to frequent under-treatment. This is frequently blamed on a lack of knowledge and pessimistic attitudes (Table 6) [51].

## 9. Advantages and Disadvantages of Traditional Techniques vs. AI

The use of AI in pain management for cancer patients represents a significant shift from traditional techniques. Both approaches have their advantages and disadvantages, which are important to consider when evaluating their effectiveness and applicability in clinical settings.

### 9.1. Traditional Techniques

• Advantages:
1. Clinical Experience: Traditional techniques are based on years of clinical experience and evidence-based practices, providing a solid foundation for pain management.
2. Personalized Care: Healthcare providers can offer personalized care by adjusting treatment based on individual patient responses and needs.
3. Human Empathy: Traditional methods allow for the human touch, which is crucial in providing comfort and support to patients experiencing pain.
4. Flexibility: Healthcare providers can adapt to unexpected situations and make decisions based on their expertise and the patient's unique circumstances.
• Disadvantages:
1. Subjectivity: Pain assessment and management can be subjective, leading to variability in treatment and outcomes.
2. Limited Data Analysis: Traditional methods may not efficiently analyze large datasets to identify patterns or predict outcomes.
3. Resource Intensive: Effective pain management can be resource-intensive, requiring significant time and effort from healthcare providers.
4. Potential for Bias: Human decision-making can be influenced by biases, which may affect treatment recommendations.

### 9.2. AI-Based Methodology

• Advantages:
1. Data-Driven Decisions: AI can analyze vast amounts of data to identify patterns and predict outcomes, potentially leading to more precise pain management strategies.
2. Consistency: AI algorithms can provide consistent and standardized assessments and treatment recommendations, reducing variability in care.
3. Efficiency: AI can process information quickly and suggest treatment options, potentially saving time and resources.
4. Personalization: Advanced AI systems can tailor treatment plans to individual patients based on their specific genetic, physiological, and psychological profiles.
5. Continuous Learning: AI systems can continuously learn from new data, improving their accuracy and effectiveness over time.
• Disadvantages:
1. Lack of Empathy: AI lacks the human element of care, which is crucial for many patients, especially those dealing with the emotional and psychological impact of cancer.
2. Ethical and Privacy Concerns: The use of AI raises ethical questions about data privacy, consent, and the potential for misuse of sensitive health information.
3. Technical Limitations: AI systems may not fully understand the complexity of human pain and the multifaceted nature of cancer pain management.
4. Dependence on Data Quality: The effectiveness of AI is highly dependent on the quality and quantity of the data it is trained on, which may be limited or biased.
5. Regulatory and Acceptance Challenges: Integrating AI into clinical practice faces regulatory hurdles and may encounter resistance from healthcare providers and patients who are skeptical of AI's role in healthcare.

In conclusion, while AI offers promising advancements in pain management for cancer patients, it is not without its challenges. Traditional techniques, grounded in clinical experience and human interaction, remain invaluable. The future of cancer pain management may lie in a hybrid approach that leverages the strengths of both traditional techniques and AI-based methodologies to provide the most effective and compassionate care possible.

## 10. Home Care Settings and Inpatient Settings

### 10.1. Home Care Settings

1. Smartphone Applications for Pain Management:
• ePAL: This smartphone application utilizes PROs and AI to optimize cancer pain management. It is particularly useful in home care settings where patients can monitor and report their pain levels regularly, allowing for timely adjustments to their pain management plan.
2. Self-Management Interventions:
• AI-driven self-management interventions can empower patients to manage their pain at home. These interventions often include educational resources, pain tracking tools, and personalized pain management plans that can be accessed and updated remotely.
3. Remote Monitoring and Support:
• AI can facilitate remote monitoring of patients' pain levels and other symptoms, allowing healthcare providers to intervene promptly if necessary. This is crucial in home care settings where patients may not have immediate access to medical facilities.

### 10.2. Inpatient Settings

1. Predictive Analytics for Pain Management:
• AI and ML tools can be used to predict pain-related outcomes and support decision-making in inpatient settings. This can help in tailoring pain management strategies to individual patient needs, ensuring more effective pain control.
2. Classifying and Managing Pain:
• AI can assist in classifying and managing pain by analyzing various data points such as patient history, current symptoms, and treatment responses. This is particularly beneficial in inpatient settings where a comprehensive and immediate assessment is often required.
3. Enhanced Decision Support Systems:
• AI-driven decision support systems can provide real-time recommendations to healthcare providers on pain management strategies. This is crucial in inpatient settings where quick and accurate decisions can significantly impact patient outcomes.
• Home Care: Focuses on patient self-management, remote monitoring, and smartphone applications that allow for continuous pain management and support.
• Inpatient Settings: Emphasizes predictive analytics, comprehensive pain classification, and enhanced decision support systems to provide immediate and tailored pain management [5].

These topics highlight the different applications of AI in pain management for cancer patients, depending on whether they are in a home care or inpatient setting.

## 11. Identifying Implicit Biases

AI offers a promising way to address these biases by analyzing large and diverse datasets, analyzing multimodal data, personalizing AI models, and data augmentation techniques [73].

### 11.1. Large and Diverse Datasets

AI models perform better and are less likely to be biased toward one group when trained on large, diverse, and representative datasets that include a wide range of demographic groups, such as different ages, genders, ethnicities, and health conditions. In pain recognition, datasets that incorporate both male and female expressions of pain, across different ethnic backgrounds, are key to improving model accuracy and fairness [74].

### 11.2. Multimodal Data

Integrating various data from different sources, including personal reports, electronic medical records, physiological and behavioral signals, and imaging data, is a promising and complementary approach to pain assessment that can increase the accuracy and sensitivity of the assessment [75].

### 11.3. Personalizing AI Models

AI can take into account personal characteristics of patients, including genetics, medical history, and imaging, to personalize pain assessment, thereby leading to the application of more personalized and efficient pain management strategies and more effective treatment [75].

### 11.4. Data Augmentation Techniques

Data augmentation is an effective technique used to increase the amount and diversity of samples to enhance ML algorithms' performance [76]. The goal of data augmentation is to artificially expand training data by generating new synthetic data points derived from existing examples. The goal of data augmentation is to artificially expand training data by generating new synthetic data points derived from existing examples. AI models can use data augmentation techniques to generate additional data for underrepresented groups to ensure that the model generalizes well across different populations [77–79].

## 12. AI and Patient Safety in Pain Management

AI is increasingly being integrated into healthcare to improve patient safety in various processes, including pain assessment, while traditional pain assessment methods can be subjective and influenced by various factors, leading to misinterpretation of pain levels; AI systems can provide objective pain detection through facial expressions, body movements, and physiological responses such as HR [74]. AI-based systems that analyze facial expressions and other behavioral cues can also identify pain in patients who cannot effectively express their pain (e.g., infants, elderly patients with dementia, or patients under sedation), leading to reduced pain and suffering [14]. Additionally, AI models, especially those using ML, can reduce human bias by providing a more standardized and unbiased assessment and ensure that pain is assessed equally across different patient groups [78, 80]. Furthermore, by detecting pain early, AI enables timely interventions, which can significantly increase patient safety by preventing pain exacerbation and related complications [11].

## 13. Conclusion

The task of managing pain in cancer patients is challenging and demanding, but the integration of AI offers a ray of hope. Recent advances in AI are beginning to change how cancer-related pain is treated and relieved as we navigate the field of oncology. AI-powered technology has the capability to provide individualized approaches for managing pain. By examining a large amount of patient data, AI can tailor pain interventions accurately by taking into account physiological signals, treatment responses, and patient-reported symptoms. This not only improves the effectiveness of pain control but also lessens adverse effects. In addition, AI allows for remote surveillance and telehealth services, enabling healthcare providers to offer timely assistance and care to cancer patients, even when they are not physically present. While challenges such as ensuring ethical AI use and protecting data privacy persist, the integration of AI with pain management in cancer care provides optimism for the future of cancer patients. This approach combines technology and empathy to enhance the quality of life for cancer patients fighting bravely. As AI progresses, our ability to reduce pain and offer comfort to those who require it will also increase. In conclusion, integrating AI with pain management in cancer care offers a significant path to more effective, patient-focused, and compassionate methods for addressing the pain associated with this challenging disease.

## Figures and Tables

**Table 1:** Advantages and drawback of both camera-based and traditional pain assessment methods.

	Advantages	Drawbacks
Camera-based method	• Noninvasive pain assessment without the need for physical contact [14]. • Pain assessment in patients who have difficulty communicating verbally, including infants or patients with dementia. • Providing objective results and reducing errors caused by individual differences and human biases [1]. • Possibility of continuous and real-time monitoring of pain [11].	• Dependence on environmental conditions, including light, viewing angle, and image quality [15] • Ignoring individual and cultural differences in pain expression • Concerns about privacy and data security • Need for large and diverse datasets

Traditional pain assessment	• Simple and accessible • Accepted standard scale • Objective and accurate assessment	• Dependence on patient reporting ability • Risk of bias and inaccuracy • Dependence on the skills and experience of the assessor • Incomplete coverage due to variation in physiological responses • Inability to recognize the severity of pain

**Table 2:** Differences between convolutional neural network (CNN) and artificial neural network (ANN) under facial expression.

Differences	Convolutional neural network (CNN)	Artificial neural network (ANN)
Architecture	• CNNs are designed specifically for processing data that has a grid-like topology, such as images. • They consist of convolutional layers, pooling layers, and fully connected layers.	• ANNs, particularly multilayer perceptrons (MLP), have a simple structure with an input layer, one or more hidden layers, and an output layer. • All neurons in a layer are fully connected to the neurons in the next layer.

Feature extraction	• CNNs automatically and adaptively learn spatial hierarchies of features from input images. • They use filters (kernels) to scan the input images and extract features, which are then used for classification tasks.	• ANNs require feature extraction to be done manually before feeding the data into the network. • The input data must be flattened to a vector, which can lead to a loss of spatial information.

Parameter sharing	• CNNs use parameter sharing, where the same filter is used across different parts of the image, reducing the number of parameters significantly.	• ANNs do not use parameter sharing, which means that the number of parameters can be very large, especially for high-resolution images.

Local connectivity	• Each neuron in a convolutional layer is only connected to a small region of the input image (receptive field), which helps in capturing local patterns and reducing the number of connections.	ANNs do not have the concept of local connectivity; each neuron in one layer is connected to every neuron in the next layer.

Pooling	• Pooling layers in CNNs reduce the spatial size of the representation, which decreases the number of parameters and computation, and helps to make the detection of features invariant to scale and orientation changes.	—

Performance	CNNs are highly effective for image recognition tasks, including facial expression recognition, because they can capture the complex patterns and features in images.	ANNs are less effective for image recognition tasks compared to CNNs because they do not consider the spatial structure of the data.

**Table 3:** Three-step “analgesic ladder” based on WHO guidelines.

Number	Type of pain	Type of analgesic used	Example
1	Mild	Nonopioids	Aspirin
2	Moderate	Weak opioids	Codeine
3	Severe	Strong opioids	Morphine

**Table 4:** Guidelines for opioid use.

Provide each opioid with a careful evaluation by adjusting the dosage until it reaches the desired effectiveness or until the patient encounters any negative reactions.
• adjust the dosage of opioids by around 25%–33% for pain that is moderately
• increase the dosage of opioids by 50%–100% for intense pain.
Administer pain medication continuously if there is consistent pain.
“Give quick-acting pain relievers for breakthrough pain (BTP)”
• The recommended BTP dosage should range from 10% to 20% of the total daily opioid dose, excluding rapid-onset fentanyl products and methadone.
• The correct dose of buprenorphine for patients who are already on methadone should be 10% of their 24-h methadone dose.
Use the simplest route of administration possible
Prevent and manage side effects: Make sure the patient is using a stool softener/stimulant to avoid constipation.
“Think about switching to a different opioid if there are unwanted side effects.”
When dealing with uncontrolled pain, it is advisable to explore different methods of administration, such as intravenous or intraspinal injections. In cases where the pain is severe and persistent in the lower half of the body, intraspinal administration may be necessary.
Consider hospitalization when pain is uncontrolled “when pain is not being managed effectively, it may be necessary to consider admission to the hospital.”
“Think about using sedation for pain and suffering that is difficult to control.”

**Table 5:** Recommendations for future predictive modeling research on opioid use disorder and overdose.

Compare various models created using machine learning and statistical methods in a fair and transparent manner to assess the advantages offered by each model accurately.
Enhance the use of external validation and future assessment to determine the effectiveness of personalized risk and monitoring models in various environments, populations, and time frames.
“Strive to gather trustworthy information on maximizing participant retention and reducing adverse events upon ending treatment by improving the delivery of OAT alongside additional interventions.”
Make sure that all predictive modeling studies follow the approved reporting standards.

**Table 6:** Challenges in pain management.

Challenges	Explanation	Solution	References
Opioid-related side effects	Opioid medications used for pain management can lead to nausea, constipation, and itching, impacting a patient's quality of life.	• Constipation: Increase dietary fiber, fluid intake, and physical activity if feasible, stimulant laxatives, osmotic laxatives, and stool softeners [52]. • Nausea: Antiemetics: Ondansetron, metoclopramide, or promethazine • Reduce opioid dose if possible or switch to an alternative opioid [53]. • Itching: Consider antihistamines (e.g., diphenhydramine, hydroxyzine) • Reduce dose or switch opioids if itching is severe [54].	Deng. Integrative medicine therapies for pain management in cancer patients [3].
Inadequate pain control	Insufficient pain relief negatively affects a patient's well-being and quality of life.	• Optimize opioid therapy: Appropriate opioid selection, dose, and route based on pain severity and patient factors. • Coping with opioid tolerance: Switch to an alternative opioid (opioid rotation) to regain effectiveness. Use NMDA receptor antagonists (e.g., ketamine, methadone) to counteract tolerance in refractory cases [55]. • Implement multimodal analgesia: Combine opioids with nonopioid analgesics. Consider interventional approaches [56]. • Consider psychological and emotional factors [57].	Scarborough and Smith. Optimal pain management for patients with cancer in the modern era [6].

Barriers to pain management	Challenges in pain management may include insufficient knowledge, poor assessment of pain, and concerns about analgesic medications.	• Educate healthcare providers on opioid prescribing, multidisciplinary, and multimodal pain management [58]. • Address patients' fears and misconceptions: Provide patient education, involve patients in pain management programs, use counseling [59]. • Use of opioid monitoring programs [60]. • Consider the biases and stigma associated with opioid use and ensure that pain relief is a priority [61].	Omoti and Omoti. Pharmacological strategies for the management of cancer pain in developing countries [49].

Psychological impact	Fear of analgesics and inadequate pain control can lead to psychological distress among cancer patients.	• Educating patients and healthcare providers about the effectiveness and proper use of painkillers can reduce fears and misconceptions [6]. • Psychological support and psychological interventions, including mindfulness techniques, can reduce patients' anxiety [62]. • Participatory decision-making and attention to patients' concerns and preferences can reduce patient anxiety [63]. • Multimodal pain management approaches and personalized pain management programs tailored to the patient's needs can lead to pain reduction and reduce patient distress [64]. • Addressing opioid-related stigma and misconceptions [65].	McKee. The challenge of cancer pain assessment [50].

Under-treatment of pain	Despite advancements, cancer patients may still not receive appropriate pain management, leading to under-treatment of pain.	• Educating patients and healthcare providers, providing clear information and educational interventions about pain management and addressing misconceptions, increases patients' confidence in using opioids for pain relief [63, 66]. • Increase the competence of healthcare providers regarding opioid use and pain management strategies [6]. • Regular pain assessment using validated tools [67]. • Multimodal pain management approaches: Combining pharmacological (opioids, NSAIDs, adjuvants) and nonpharmacological (physical therapy, acupuncture, psychological interventions) methods [68]. • Enhancing access to pain management services [66].	Makhlouf et al. Managing pain in people with cancer-a systematic review of the attitudes and knowledge of professionals, patients, caregivers and public [51].

Invasive procedures underutilization	Invasive interventional procedures for pain management in cancer patients may be underutilized despite their effectiveness.	• Increase awareness and education among healthcare providers and patients about the benefits and address concerns and misconceptions [69, 70]. • Improving access to specialized and invasive pain services [71].	Bhaskar. Interventional pain management in patients with cancer-related pain [72].

## Data Availability

Datasets generated during the current study are available from the corresponding author upon reasonable request.

## References

[1] Gkikas S., Tsiknakis M. (2023). Automatic Assessment of Pain Based on Deep Learning Methods: A Systematic Review. *Computer Methods and Programs in Biomedicine*.

[2] Mercadante S., Coluzzi F. (2021). Factors Influencing Pain Expression in Patients With Cancer: An Expert Opinion. *Pain and therapy*.

[3] Deng G. (2019). Integrative Medicine Therapies for Pain Management in Cancer Patients. *The Cancer Journal*.

[4] Bhatnagar S., Gupta M. (2015). Evidence-based Clinical Practice Guidelines for Interventional Pain Management in Cancer Pain. *Indian Journal of Palliative Care*.

[5] van den Beuken-van Everdingen M. H. J., Hochstenbach L. M., Joosten E. A., Tjan-Heijnen V. C., Janssen D. J. (2016). Update on Prevalence of Pain in Patients With Cancer: Systematic Review and Meta-Analysis. *Journal of Pain and Symptom Management*.

[6] Scarborough B. M., Smith C. B. (2018). Optimal Pain Management for Patients With Cancer in the Modern Era. *CA: A Cancer Journal for Clinicians*.

[7] Yates P. M., Edwards H. E., Nash R. E. (2002). Barriers to Effective Cancer Pain Management: A Survey of Hospitalized Cancer Patients in Australia. *Journal of Pain and Symptom Management*.

[8] Borna S., Haider C. R., Maita K. C. (2023). A Review of Voice-Based Pain Detection in Adults Using Artificial Intelligence. *Bioengineering*.

[9] Cascella M., Schiavo D., Cuomo A. (2023). Artificial Intelligence for Automatic Pain Assessment: Research Methods and Perspectives. *Pain Research and Management*.

[10] Tavakolian M., Hadid A. (2019). A Spatiotemporal Convolutional Neural Network for Automatic Pain Intensity Estimation From Facial Dynamics. *International Journal of Computer Vision*.

[11] Wu C.-L., Liu S.-F., Yu T.-L. (2022). Deep Learning-Based Pain Classifier Based on the Facial Expression in Critically Ill Patients. *Frontiers of Medicine*.

[12] Reichard B., Schrumpf F., Anders F., Bode K., Fuchs M. (2022). Camera-Based Pain Assessment During Surgical Interventions. *Current Directions in Biomedical Engineering*.

[13] Salama V., Godinich B., Geng Y. (2024). Artificial Intelligence and Machine Learning in Cancer Pain: A Systematic Review. *Journal of Pain and Symptom Management*.

[14] Tuttle A. H., Molinaro M. J., Jethwa J. F. (2018). A Deep Neural Network to Assess Spontaneous Pain From Mouse Facial Expressions. *Molecular Pain*.

[15] Hammal Z., Cohn J. F. Automatic Detection of Pain Intensity.

[16] Pise A. A., Alqahtani M. A., Verma P. (2022). Methods for Facial Expression Recognition With Applications in Challenging Situations. *Computational Intelligence and Neuroscience*.

[17] Zamzmi G., Paul R., Salekin M. S. (2019). Convolutional Neural Networks for Neonatal Pain Assessment. *IEEE Transactions on Biometrics, Behavior, and Identity Science*.

[18] Hosseini E., Fang R., Zhang R. Convolution Neural Network for Pain Intensity Assessment From Facial Expression.

[19] Bargshady G., Soar J., Zhou X., Deo R. C., Whittaker F., Wang H. A Joint Deep Neural Network Model for Pain Recognition From Face.

[20] Monwar M. M., Rezaei S. Pain Recognition Using Artificial Neural Network.

[21] Shuang Y., Liangbo G., Huiwen Z. (2024). Classification of Pain Expression Images in Elderly with Hip Fractures Based on Improved ResNet50 Network. *Frontiers of Medicine*.

[22] Wang L., Wang Z., Xu A., Liu S. A Generative Adversarial Network-Based Approach for Facial Pain Assessment.

[23] Rojas R. F., Romero J., Lopez-Aparicio J., Ou K. L. Pain Assessment Based on fNIRS Using Bi-LSTM RNNs.

[24] Cascella M., Schiavo D., Cuomo A. (2023). Artificial Intelligence for Automatic Pain Assessment: Research Methods and Perspectives. *Pain Research and Management*.

[25] Ewers A., Gnass I. (2018). [Painapp-Mobile Pain Monitoring in the Home Care Setting]. *Schmerz, Der*.

[26] Nagireddi J., Vyas A., Sanapati M., Soin A., Manchikanti L. (2022). The Analysis of Pain Research Through the Lens of Artificial Intelligence and Machine Learning. *Pain Physician*.

[27] Fernandez Rojas R., Brown N., Waddington G., Goecke R. (2023). A Systematic Review of Neurophysiological Sensing for the Assessment of Acute Pain. *npj Digital Medicine*.

[28] Werner P., Lopez-Martinez D., Walter S., Al-Hamadi A., Gruss S., Picard R. W. (2022). Automatic Recognition Methods Supporting Pain Assessment: A Survey. *IEEE Transactions on Affective Computing*.

[29] Schneider R. L., Arch J. J., Wolitzky-Taylor K. B. (2015). The State of Personalized Treatment for Anxiety Disorders: A Systematic Review of Treatment Moderators. *Clinical Psychology Review*.

[30] Jackson S. E., Chester J. D. (2015). Personalised Cancer Medicine. *International Journal of Cancer*.

[31] Manworren R. C. (2015). Multimodal Pain Management and the Future of a Personalized Medicine Approach to Pain. *AORN Journal*.

[32] Parikh R. B., Gdowski A., Patt D. A., Hertler A., Mermel C., Bekelman J. E. (2019). Using Big Data and Predictive Analytics to Determine Patient Risk in Oncology. *American Society of Clinical Oncology educational book. American Society of Clinical Oncology. Annual Meeting*.

[33] Chua I. S., Gaziel‐Yablowitz M., Korach Z. T. (2021). Artificial Intelligence in Oncology: Path to Implementation. *Cancer Medicine*.

[34] Massaad E., Fatima N., Hadzipasic M., Alvarez-Breckenridge C., Shankar G. M., Shin J. H. (2019). Predictive Analytics in Spine Oncology Research: First Steps, Limitations, and Future Directions. *Neurospine*.

[35] Cova T. F., Bento D. J., Nunes S. C. (2019). Computational Approaches in Theranostics: Mining and Predicting Cancer Data. *Pharmaceutics*.

[36] Gaedke Nomura A. T., de Abreu Almeida M., Johnson S., Pruinelli L. (2021). Pain Information Model and Its Potential for Predictive Analytics: Applicability of a Big Data Science Framework. *Journal of Nursing Scholarship*.

[37] Bakitas M., Cheville A. L., Mulvey T. M., Peppercorn J., Watts K., Dionne-Odom J. N. (2021). Telehealth Strategies to Support Patients and Families Across the Cancer Trajectory. *American Society of Clinical Oncology Educational Book. American Society of Clinical Oncology. Annual Meeting*.

[38] Li J., Liu Y., Jiang J., Peng X., Hu X. (2021). Effect of Telehealth Interventions on Quality of Life in Cancer Survivors: A Systematic Review and Meta-Analysis of Randomized Controlled Trials. *International Journal of Nursing Studies*.

[39] Hwang N.-K., Jung Y.-J., Park J.-S. (2020). Information and Communications Technology-Based Telehealth Approach for Occupational Therapy Interventions for Cancer Survivors: A Systematic Review. *Healthcare*.

[40] Cascella M., Coluccia S., Monaco F. (2022). Different Machine Learning Approaches for Implementing Telehealth-Based Cancer Pain Management Strategies. *Journal of Clinical Medicine*.

[41] Sams M., Eggerth A., Hayn D., Veeranki S., Schreier G. (2019). Predictive Modelling and Its Visualization for Telehealth Data—Concept and Implementation of an Interactive Viewer. *Studies in Health Technology and Informatics*.

[42] Schug S. A., Chandrasena C. (2015). Pain Management of the Cancer Patient. *Expert Opinion on Pharmacotherapy*.

[43] Brozović G., Lesar N., Janev D., Bošnjak T., Muhaxhiri B. (2022). Cancer Pain and Therapy. *Acta Clinica Croatica*.

[44] Bharat C., Hickman M., Barbieri S., Degenhardt L. (2021). Big Data and Predictive Modelling for the Opioid Crisis: Existing Research and Future Potential. *The Lancet Digital Health*.

[45] Shanthanna H., Ladha K. S., Kehlet H., Joshi G. P. (2021). Perioperative Opioid Administration: A Critical Review of Opioid-Free Versus Opioid-Sparing Approaches. *Anesthesiology*.

[46] Paice J. A., Bohlke K., Barton D. (2023). Use of Opioids for Adults With Pain From Cancer or Cancer Treatment: ASCO Guideline. *Journal of Clinical Oncology*.

[47] Howard A., Brant J. M. (2019). *Pharmacologic Management of Cancer Pain. Seminars in Oncology Nursing*.

[48] Gadhia S., Richards G. C., Marriott T., Rose J. (2023). Artificial Intelligence and Opioid Use: A Narrative Review. *BMJ Innovations*.

[49] Omoti A. E., Omoti C. E. (2007). Pharmacological Strategies for the Management of Cancer Pain in Developing Countries. *Pharmacy Practice*.

[50] McKee R. (2018). The Challenge of Cancer Pain Assessment. *Ulster Medical Journal*.

[51] Makhlouf S. M., Pini S., Ahmed S., Bennett M. I. (2020). Managing Pain in People with Cancer—A Systematic Review of the Attitudes and Knowledge of Professionals, Patients, Caregivers and Public. *Journal of Cancer Education*.

[52] Pergolizzi J. V., Lequang J. A., Passik S., Coluzzi F. (2019). Using Opioid Therapy for Pain in Clinically Challenging Situations. Questions for Clinicians. *Minerva Anestesiologica*.

[53] Benyamin R., Trescot A. M., Datta S. (2008). Opioid Complications and Side Effects. *Pain Physician*.

[54] Ganesh A., Maxwell L. G. (2007). Pathophysiology and Management of Opioid-Induced Pruritus. *Drugs*.

[55] Mercadante S., Adile C., Tirelli W., Ferrera P., Penco I., Casuccio A. (2021). Barriers and Adherence to Pain Management in Advanced Cancer Patients. *Pain Practice*.

[56] Els C., Jackson T. D., Kunyk D. (2017). Adverse Events Associated With Medium‐and Long‐Term Use of Opioids for Chronic Non‐Cancer Pain: An Overview of Cochrane Reviews. *Cochrane Database of Systematic Reviews*.

[57] Garland E. L., Brintz C. E., Hanley A. W. (2020). Mind-Body Therapies for Opioid-Treated Pain: A Systematic Review and Meta-Analysis. *JAMA Internal Medicine*.

[58] Eccleston C., Fisher E., Howard R. F. (2021). Delivering Transformative Action in Paediatric Pain: A Lancet Child & Adolescent Health Commission. *The Lancet Child & Adolescent Health*.

[59] Matthias M. S., Krebs E., Bergman A., Coffing J., Bair M. (2014). Communicating About Opioids for Chronic Pain: A Qualitative Study of Patient Attributions and the Influence of the Patient–Physician Relationship. *European Journal of Pain*.

[60] Volkow N. D., McLellan A. T. (2016). Opioid Abuse in Chronic Pain—Misconceptions and Mitigation Strategies. *New England Journal of Medicine*.

[61] Cheetham A., Picco L., Barnett A., Lubman D. I., Nielsen S. (2022). The Impact of Stigma on People With Opioid Use Disorder, Opioid Treatment, and Policy. *Substance Abuse and Rehabilitation*.

[62] Syrjala K. L., Jensen M. P., Mendoza M. E., Yi J. C., Fisher H. M., Keefe F. J. (2014). Psychological and Behavioral Approaches to Cancer Pain Management. *Journal of Clinical Oncology*.

[63] Oldenmenger W. H., Geerling J. I., Mostovaya I. (2018). A Systematic Review of the Effectiveness of Patient-Based Educational Interventions to Improve Cancer-Related Pain. *Cancer Treatment Reviews*.

[64] Kalso E., Edwards J. E., Moore A. R., McQuay H. J. (2004). Opioids in Chronic Non-Cancer Pain: Systematic Review of Efficacy and Safety. *Pain*.

[65] Harsanyi H., Cuthbert C., Schulte F. (2023). The Stigma Surrounding Opioid Use as a Barrier to Cancer-Pain Management: An Overview of Experiences With Fear, Shame, and Poorly Controlled Pain in the Context of Advanced Cancer. *Current Oncology*.

[66] Yazdani S., Abdi S. (2014). Brief Review: Pain Management for Cancer Survivors: Challenges and Opportunities. *Canadian Journal of Anesthesia/Journal canadien d’anesthésie*.

[67] Wiffen P. J., Wee B., Derry S., Bell R. F., Moore R. A. (2017). Opioids for Cancer Pain—An Overview of Cochrane Reviews. *Cochrane Database of Systematic Reviews*.

[68] Kettyle G. (2023). Multidisciplinary Approach to Cancer Pain Management. *Ulster Medical Journal*.

[69] Tay W., Ho K. Y. (2009). The Role of Interventional Therapies in Cancer Pain Management. *Annals Academy of Medicine Singapore*.

[70] Manchikanti L., Kaye A. M., Knezevic N. N. (2023). Comprehensive, Evidence-Based, Consensus Guidelines for Prescription of Opioids for Chronic Non-Cancer Pain From the American Society of Interventional Pain Physicians (ASIPP). *Pain Physician*.

[71] Gibek E. K., Schulman-Green D. J. (2021). Increasing Access to Interventional Pain Management Therapies for Palliative Care Patients With Cancer Through Referral System Improvement (F425D). *Journal of Pain and Symptom Management*.

[72] Bhaskar A. (2020). Interventional Pain Management in Patients With Cancer-Related Pain. *Postgraduate Medicine*.

[73] Li H., Moon J. T., Shankar V., Newsome J., Gichoya J., Bercu Z. (2024). Health Inequities, Bias, and Artificial Intelligence. *Techniques in Vascular and Interventional Radiology*.

[74] Lucey P., Cohn J., Kanade T., Saragih J., Ambadar Z., Matthews I. (2010). The Extended Cohn-Kanade Dataset (CK+): A Complete Dataset for Action Unit and Emotion-Specified Expression.

[75] El-Tallawy S. N., Pergolizzi J. V., Vasiliu-Feltes I. (2024). Incorporation of “Artificial Intelligence” for Objective Pain Assessment: A Comprehensive Review. *Pain and Therapy*.

[76] Al-Qerem A. (2020). An Efficient Machine-Learning Model Based on Data Augmentation for Pain Intensity Recognition. *Egyptian Informatics Journal.*.

[77] Al-Nafjan A., Alshehri H., Aldayel M. (2025). Objective Pain Assessment Using Deep Learning Through EEG-Based Brain–Computer Interfaces. *Biology*.

[78] Mehrabi N., Morstatter F., Saxena N., Lerman K., Galstyan A. (2021). A Survey on Bias and Fairness in Machine Learning. *ACM Computing Surveys*.

[79] Ghane G., Cheraghi M. A., Pashaeypoor S., Najafi F. (2021). Concept Analysis of the Four‐Season‐Symphony of Intellectuality–Spirituality–Ethics–Esthetics (FSS: I SEA) in Nursing Research. *Nursing Forum*.

[80] Ghane G., Farahani M. A., Seyedfatem N., Haghani H. (2017). Effect of Problem-Focused Coping Strategies on the Quality of Life in Family Caregivers of Hemodialysis Patients. *Koomesh*.

